# Prevalent and Severe Conditions That Compromise the Welfare of Shelter Dogs: Opinions from the Taiwanese Experts

**DOI:** 10.3390/ani15040592

**Published:** 2025-02-18

**Authors:** Yu-Hsin Chen, Ching-I Chen, Chen-Yan Lin, Kendy Tzu-yun Teng

**Affiliations:** 1 Department of Veterinary Medicine, National Chung Hsing University, No. 145, Xingda Rd., South Dist., Taichung City 40227, Taiwan; yhchen1806@gmail.com (Y.-H.C.); 910711jean@gmail.com (C.-I.C.); elericlin9131@gmail.com (C.-Y.L.); 2 The iEGG and Animal Biotechnology Research Center, National Chung Hsing University, No. 145, Xingda Rd., South Dist., Taichung City 40227, Taiwan

**Keywords:** welfare conditions, shelter dog, public animal shelters, Five Domains, Delphi approach, expert consensus, animal welfare, Taiwan

## Abstract

The welfare of shelter dogs is a growing concern, particularly in regions like Taiwan, where public animal shelters (PASs) often face overcrowding. This study aimed to identify and prioritise welfare conditions (WCs) affecting shelter dogs in Taiwan’s PASs, offering insights into their impacts on population and individual welfare. A modified Delphi approach engaged 14 experts to evaluate the prevalence, severity, and duration of 59 potential WCs using the Five Domains model. The welfare impact of each WC on individual dogs was assessed as the product of severity and duration, with WCs scoring a median of 3 or higher considered critical. Of the 59 identified WCs, 49 were deemed critical. The most prevalent WCs were “limited access to toys” (median = 4.00), “insufficient foraging opportunities” (median = 4.00), and “intermittent, excessive barking noise” (median = 4.00). The most impactful WCs for individual dogs were “depressive disorders” (median of the product of duration and severity scores = 16.00), “fear-related or anxiety disorders” (median = 15.00), and “limited space” (median = 15.00). The Physical Environment Domain and the Behavioural Interactions Domain were highlighted as having the most significant and prolonged negative effects on welfare. This study provides a comprehensive WC list for shelter dogs, guiding future welfare improvements and resource allocation strategies.

## Simple Summary

Shelter dogs often face difficult living conditions, especially in overcrowded public animal shelters like those in Taiwan. This study aimed to identify the important welfare conditions (WCs) that negatively affect shelter dogs. Using expert opinion and a structured evaluation method, 59 potential WCs were reviewed and ranked based on their prevalence, severity, and duration. From these, 49 were considered critical for improving the welfare of shelter dogs. The most common conditions included “limited access to toys”, “insufficient foraging opportunities”, and “intermittent, excessive barking noise”. The most serious problems for individual dogs were “depressive disorders“, “fear-related or anxiety disorders“, and “limited space”. WCs related to the Physical Environment Domain and the Behavioural Interactions Domain were relatively common for shelter dogs, compared to the Health Domain or the Nutrition Domain. This research provides a detailed list of WCs that can help shelters prioritise actions to improve living conditions for dogs. By focusing on the most impactful conditions, shelters can better allocate resources and create healthier, more comfortable environments for dogs awaiting adoption.

## 1. Introduction

The issue of free-roaming dogs represents a significant challenge worldwide [1,2], adversely affecting public health, wildlife conservation, and livestock production [3,4,5]. The global population of dogs is estimated at approximately 0.7–1 billion, with 76–83% categorised as free-roaming [6]. Dog sheltering is among the most widely adopted strategies to address this issue [7]. While public shelters in some regions implement catch–neuter–return (CNR) (i.e., trap–neuter–return) programmes to reduce the reproduction of free-roaming dogs and/or prevent prolonged confinement [8,9], many others adopt the policy of retention until they are adopted or die, whether assisted or unassisted [9,10,11]. Precise data on the number of dogs entering animal shelters or rescue organisations globally is difficult to obtain. However, according to statistics from Shelter Animals Count for the first half of 2024, as many as 1.33 million dogs entered shelters in the United States alone [12].

The primary purpose of animal shelters is to provide temporary care before relocating or rehoming animals. However, shelter infrastructure and management practices often fail to adequately address shelter animals’ physiological, mental, and social needs [13,14]. In Taiwan, the implementation of the “no-kill” policy in 2017 prohibits the euthanasia of animals for free-roaming or shelter population management [15,16]. This policy has resulted in more animals entering shelters than leaving in at least one-third of the public animal shelters (PASs) in Taiwan [17]. When there is a substantial increase in shelter intake without a corresponding rise in adoptions, lengths of stay are prolonged [17,18] and shelters likely become overcrowded, surpassing their capacity for care [19,20]. In the PASs in Taiwan, some animals, especially dogs, may stay for an extended period (i.e., ≥3 months), leading to a population of long-term shelter residents whose welfare is challenging to maintain [21,22].

For shelter dogs, one of the most significant welfare challenges is the lack of environmental predictability and controllability, particularly for new arrivals [23,24]. These dogs are exposed to novel stimuli, including kennel environments, noise, odours, restraint, and interactions with unfamiliar staff, visitors, and other animals [14,25,26,27,28]. Additionally, they face a heightened risk of infection due to overcrowding and stress-induced immunosuppression [29]. Many studies have investigated the welfare of shelter dogs using an animal-based approach [30,31] through physiological and behavioural assessments. Physiological assessments, including cortisol levels [32,33,34], heart rate variability [35,36,37], and oxidative stress parameters [38,39]. Various behavioural assessments, such as quality of life assessment and Shelter Quality Protocol (SQP), have also been applied in many studies to evaluate their welfare [40,41]. However, understanding the impact of environmental conditions and resource allocation on shelter dogs is equally critical. Effective use of limited shelter resources requires identifying and addressing severe welfare conditions (WCs) to promote the welfare of these animals more effectively.

The task of identifying important WCs in animal shelters requires two essential steps: identification and prioritisation. Many animal welfare frameworks, such as the Five Domains [42] and the Welfare Quality model [43], have been widely applied to facilitate the identification and assessment of WCs in various species of animals [44,45,46,47]. The Five Domains outline five key aspects of animal welfare. Four of these are physical and physiological domains: Nutrition, Physical Environment, Health, and Behavioural Interactions. The fifth domain, Mental State, is interconnected with all the physical and physiological domains, reflecting their combined influence on overall welfare [42]. However, to our knowledge, the Five Domains framework has not yet been applied to shelter animal settings. The SQP, derived from the Welfare Quality model, focuses on welfare assessment for shelter animals by addressing good feeding, good housing, good health, and appropriate behaviours [43]. The SQP incorporates both resource- and management-based measures as well as animal-based indicators [48]. However, it does not aim to assess the severity of individual WCs. Given that shelter animals often face multiple WCs simultaneously, quantifying the negative welfare impact of individual impacts remains a significant challenge.

Expert opinion is a common method for assessing the severity of WCs [49,50,51], with the Delphi approach being a particularly prominent example [49,51]. This iterative process involves surveys and discussions to achieve expert consensus, reflecting a broader and more accurate perspective than individual opinions [52]. The Delphi approach involves iterative rounds of surveys and discussions to achieve consensus among experts, although no standardised procedure for its implementation currently exists [53]. This approach has been applied to identify welfare priorities for various species, including horses [53,54], rabbits [55], fish [56], laboratory mice [57], and pet dogs [58,59]. It has also been used to determine the weighting of different welfare measures within the SQP. However, the Delphi approach has not yet been applied to assess the severity of a comprehensive list of WCs for shelter dogs [60].

This study aims to identify the important WCs faced by dogs in PASs in Taiwan. A comprehensive evaluation based on the Five Domains model was conducted to generate a list of WCs for shelter dogs [42]. A modified Delphi approach was employed, involving Taiwanese experts and scholars from relevant fields. Participants completed questionnaires and engaged in focus group discussions to identify and prioritise important WCs. The findings of this study will be shared with the local animal protection agency in Taiwan to enhance their understanding of the impact of WCs on shelter dogs and support efforts to improve animal welfare.

## 2. Materials and Methods

### 2.1. Ethics Approval

Ethics approval for study procedures was obtained from the National Cheng Kung University Human Research Ethics Committee (NCKU HREC-E-112-639-2).

### 2.2. Identification of Prioritised Welfare Conditions

#### 2.2.1. Initial List of Welfare Conditions

The initial list of potential WCs for dogs was developed using existing knowledge, a comprehensive literature review, and field observations at PASs. The literature search was conducted on PubMed with the following keywords: (animal welfare OR welfare) AND (shelter OR kennel) AND (dog OR canine OR puppy OR puppies). Titles and abstracts were manually reviewed to identify relevant situations affecting the welfare of shelter dogs. WCs were defined as external or internal factors that directly contribute to negative welfare outcomes for shelter dogs. The list of WCs was then modified to reflect the situations in Taiwan’s shelters through visiting PASs. The WCs in the list were categorised into one of the four physical and functional domains of the “Five Domains” welfare model: Nutrition, Physical Environment, Health, and Behavioural Interactions [42].

#### 2.2.2. Questionnaire Design

The questionnaire was structured into three modules. The first module explained the aims and scope of the questionnaire. The second module involved scoring each potential WC, presented in random order, on three indicators: prevalence, severity, and duration. These indicators were measured using a 6-point (0–5) Likert scale. Prevalence was defined as “the average proportion of dogs affected by a WC at any given time in recent years in the PASs”, with a score of 0 indicating a prevalence of 0.0%, 1 indicating greater than 0.0% to 20.0%, 2 indicating greater than 20.0% to 40.0%, 3 indicating greater than 40.0% to 60.0%, 4 indicating greater than 60.0% to 80.0%, and 5 indicating greater than 80.0% to 100.0%. Severity was defined as “the average negative impact of a WC on a dog’s affective state”. From 0 to 5, the score denoted (a) no impact, (b) the mildest impact, (c) mild impact, (d) moderate impact, (e) severe impact, and (f) the most severe impact on the dog’s affective state. Duration was defined as “the average length of time a WC (i.e., condition or disease) persists in an individual at the PASs”. From 0 to 5, the score indicated a duration of (a) less than one week, (b) one week to one month, (c) one to three months, (d) three to six months, (e) six to twelve months, and (f) more than one year, respectively. The final module asked participants to propose any WCs that were not initially presented in our list. The proposed WCs were then evaluated by three team members (i.e., Ching-I Chen, Chen-Yan Lin, and Kendy Tzu-yun Teng), followed by a discussion. The newly added WCs were then scored by the experts during the focus group discussion.

### 2.3. Welfare Condition Prioritisation

#### 2.3.1. Recruitment of Experts

Leveraging the researchers’ network, 14 experts were recruited from various stakeholder groups, including 10 veterinarians (4 shelter veterinarians, 4 veterinarians with profound knowledge of canine behaviour, and 2 veterinary internists), 1 dog trainer, and 3 animal welfare experts. Shelter veterinarians were eligible if they had worked at a PAS for over one year. Veterinarians with profound knowledge of canine behaviour, veterinary internists, and dog trainers were required to have more than three years of experience. Animal welfare experts were required to have a master’s or PhD degree in the relevant field.

#### 2.3.2. Modified Delphi Approach

In this study, a modified Delphi approach was used to identify Taiwanese experts’ opinions on WCs. Firstly, the experts were asked to complete the aforementioned questionnaire on Google Sheets (Mountain View, CA, USA) within one week before a focus group discussion. Two focus groups with 7 experts each were carried out due to practical considerations for experts’ availability. For each WC, any instance where the prevalence, severity, or duration had a standard deviation of scores ≥ 1.25 among the seven experts was selected for discussion in the focus group meeting. The focus group meetings, each approximately 3 h long, were conducted via Google Meet (Mountain View, CA, USA). The minutes of both meetings were sent to all experts within 5 days, and the experts were asked to review the minutes and modify their scores if they found them appropriate.

### 2.4. Data Analysis

Both the encoding of questionnaire responses and the descriptive statistics [i.e., median and interquartile range] were conducted using Google Sheets. Using the final scores provided after the focus group, a WC was selected as an “important WC” if any of the prevalence, severity, or duration had a median ≥ 3. The products of the medians of duration and severity scores were calculated to represent the impact of the WC on individual dogs and considered “important” if ≥9.

## 3. Results

### 3.1. Welfare Conditions List

A total of 58 WCs were identified for the questionnaire based on background knowledge, observations at PASs, and a systematic review of 388 results from the literature search (Table 1). All 58 WCs were included in the questionnaire for veterinarians, whereas only 42 WCs, excluding most health-related conditions, were included in the version for non-veterinarians. Following expert suggestions during the first round of the survey, the category “fear, anxiety, or discomfort-inducing stimuli resulting from humans (including staff and visitors)” was refined into two specific categories: “fear, anxiety, or discomfort-inducing stimuli caused by staff and volunteers” and “fear, anxiety, or discomfort-inducing stimuli caused by visitors”. This refinement increased the total number of WCs to 59, as presented in Table 1.

### 3.2. Demographics of the Experts and Research Implementation

A total of 14 experts participated in this study, with a median age of 40 years (range: 27–63), and the majority were female (*n* = 10; 71.4%). They included 10 veterinarians (71.4%), three animal welfare experts (21.4%), and one dog trainer (7.1%). Geographically, the experts were based in Northern Taiwan (*n* = 5; 35.7%), Central Taiwan (*n* = 3, 21.4%), Southern Taiwan (*n* = 3, 21.4%), Eastern Taiwan (*n* = 1, 7.1%), the United States (*n* = 1, 7.1%), and other regions (*n* = 1, 7.1%). The response rate for the online questionnaire was 100.0% in both rounds. The number of items (i.e., any of the prevalence, severity, or duration) discussed was 59 in the first focus group meeting and 73 in the second focus group meeting.

### 3.3. Prioritised Welfare Conditions List

In total, 49 out of 59 of the WCs were considered important WCs (Table A1). Among these, 16 (27.1%), 39 (66.1%), and 27 (45.8%) WCs had a median score of 3 or higher for prevalence, severity, and duration, respectively. In terms of welfare impact on the population, 16 WCs were considered to have a high prevalence, defined as affecting more than 40.0% of dogs in PASs (Table 2). Of these 16 WCs, 6 were classified under the Physical Environment Domain and 10 under the Behavioural Interaction Domain. In the evaluation of welfare impact on individuals, 20 WCs were considered important for shelter dogs in PASs (Table 3). This included 0 out of 5 (0.0%) of the WCs in the Nutrition Domain, 6 out of 12 (50.0%) WCs in the Physical Environment Domain, 9 out of 28 (32.1%) of the WCs in the Health Domain, and 5 out of 14 (35.7%) in the Behavioural Interaction Domain. This indicates that experts perceived environmental WCs as having a more severe and/or prolonged impact on the welfare of shelter dogs.

## 4. Discussion

In this study, we identified 59 WCs affecting shelter dogs across the “Five Domains,” with 49 deemed important based on expert opinions. The Physical Environment Domain and the Behavioural Interactions Domain were considered important for both individual- and population-level welfare compared to those in the Nutrition Domain and the Health Domain. This study provides a comprehensive list of WCs that can serve as a framework for assessing the welfare of dogs housed in animal shelters. By prioritising these WCs through expert opinion, targeted improvements can be implemented in public and private animal shelters in Taiwan.

The shelter environment plays a critical role in determining whether dogs feel calm, relaxed, and comfortable. Dogs lacking access to restful spaces or exposed to overstimulating stimuli may increase their stress level in the shelter [96]. Experts indicated that barking noise, overstimulating odours, and lacking suitable rest materials are prevalent conditions in PASs in Taiwan, potentially hindering dogs’ ability to achieve quality sleep and rest. Barking noise has been shown to increase stress levels of shelter dogs, which may reduce resting behaviours [14,97,98]. In animal shelters, it often reaches 100 decibels, which may impair shelter dogs’ hearing [14,99]. Shelter dogs often experience elevated stress levels due to the environment, which causes resting behaviour to decrease [97]. Similarly, olfactory overstimulation from strong shelter odours might also elevate stress levels [100], although further research is needed to confirm this effect. Interestingly, certain fragrances, such as coconut and ginger, have demonstrated calming effects on shelter dogs [101], suggesting that the odour likely affects the welfare of shelter dogs. In addition to auditory and olfactory stressors, a lack of suitable substrates may also prevent dogs from obtaining adequate rest. Dogs prefer soft bedding materials [102,103], while shelter floors or cage bottoms are often cold, wet, or uncomfortable. Limited space may further compromise sleep quality by restricting movement or forcing dogs to rest near their excrement [104,105].

In shelters with limited resources, priority is often placed on meeting the basic physiological needs of dogs [106]. Even basic enrichment might be unavailable for shelter dogs [107]. Barren environments may increase stress levels and the likelihood of behavioural problems in shelter dogs [108]. While the most effective types of enrichment may vary depending on individual dogs, previous studies suggest that music, toys, pheromones, and conspecific play can significantly reduce stress levels and stress-related behaviours [91,109,110,111]. Shelter dogs frequently lack opportunities for positive social interactions with humans and other dogs. Experts even indicated that fear, anxiety, or discomfort caused by shelter staff and volunteers is common [112,113]. Shelter dogs in PASs in Taiwan are often cohoused due to limited space. While cohousing can be beneficial for dogs [95,114], improper pairing or grouping can lead to fear, anxiety, aggression, and even injuries. Many shelters in the United States implement individual housing paired with structured group play sessions. This approach has demonstrated several positive outcomes, including reductions in abnormal behaviours and agonistic interactions and increased adoption rates [115,116,117]. These findings highlight the importance of enrichment and appropriate social opportunities in improving the welfare of shelter dogs.

Approximately half of the 59 WCs identified in this study fall under the Health Domain. Notably, despite the lack of standardised diagnostic criteria for psychological conditions such as depressive disorders and fear-related or anxiety disorders, these two conditions are ranked as having the greatest negative welfare impact on individual shelter dogs in PASs in Taiwan. Dogs with anxiety disorders exhibit stress-related behaviours such as hypervigilance, increased reactivity, trembling, and hypersalivation without the presence of the trigger stimuli [118]. Anxiety disorders in dogs can be generalised or specific. Generalised anxiety involves anxiety-related behaviours in three or more distinct circumstances, while specific anxiety is limited to a particular situation, both occurring without the presence of triggering stimuli [119,120]. Interestingly, while the diagnosis of anxiety disorders in dogs is relatively common in veterinary practice [121], it remains uncertain whether dogs can truly experience depressive disorders. MacLellan et al. (2021) reviewed existing literature to explore whether animals are capable of experiencing depressive states, which are typically characterised by low activity levels and reduced interest in the external environment (anhedonia) [122]. The review found evidence suggesting that animals, including dogs, are indeed capable of clinical depression. However, depression-like conditions in dogs may not manifest in the same behaviours observed in humans with clinical depression. For example, shelter dogs that spend extended periods “awake but motionless”, which could be interpreted as an anhedonic behaviour, did not exhibit reduced interest in food-filled toys, another anhedonic behaviour [75]. Instead, they showed the opposite. These findings emphasise the need to avoid anthropomorphising dog behaviours when assessing their affective states. Further research is required to better understand how depressive states manifest in dogs and to develop appropriate diagnostic criteria. Regardless, it is essential to identify and address shelter dogs’ behaviours indicative of negative affective states as early as possible. Behavioural problems significantly reduce the likelihood of adoption and increase the risk of dogs being returned after adoption [123,124,125].

Our study highlights the importance of considering both the severity and duration of diseases or WCs when evaluating their impact on animal welfare. Diseases often exert a profound negative effect, as evidenced by our findings, where 22 out of 28 WCs in the Health Domain had an average severity score of 3 or higher. However, when considering duration, only 10 WCs in the same domain received an average score of 3 or higher. This discrepancy may be attributed to the nature of fatal infectious diseases in shelters, where limited resources often necessitate euthanasia upon diagnosis [29,126]. In contrast, WCs in the Physical Environment Domain tend to persist longer if management practices remain unchanged, yet their severity is typically less acute than that of diseases. Fifty percent of the WCs in the Physical Environment Domain were considered important for their impact on individual dogs by experts, compared to 32.1% in the Health Domain. This finding underscores the critical role of duration in estimating the welfare impact of WCs. Relying on an assessment framework that considers only severity, without accounting for duration, risks underestimating the true impact of WCs. The length of time a WC affects an animal is as crucial as the intensity of its effects, as also shown in the Welfare-Adjusted Life Years method [127]. Thus, both dimensions must be integrated into a comprehensive animal welfare assessment framework.

We believe that the list of WCs can serve as a valuable tool for shelters to assess the welfare of shelter dogs. Our recommended assessment methods for each WC are outlined in Table A2. Assessment methods vary depending on the specific condition and include direct observation, instrument-based measurements, analysis of government open data, staff interviews, and reviews of medical records [87]. To effectively utilise the WC list, it is crucial to train observers to recognise stress-related behaviours and physiological indicators associated with stress or illness in dogs [48,128]. Additionally, instruments such as temperature data loggers [129], decibel metres [87], gas detectors [130,131,132,133], and lux metres [134] should be employed to objectively evaluate environmental conditions. As continuous observation is not always feasible, interviews with shelter staff can provide insights into daily care routines and medical histories, offering a more comprehensive understanding of shelter operations [135,136,137]. We recommend that shelters maintain detailed records of environmental parameters and the physiological conditions and behavioural expressions of individual dogs. This WC list can be used periodically—every three or six months, for example—to evaluate the welfare of dogs in the shelter. Based on these assessments, resources can be strategically allocated to address identified conditions and improve the overall welfare of the dogs.

We acknowledge several limitations of the current study. First, the geographical representation of experts was primarily centred on Taiwan, which may limit the generalisability of the findings to regions with different shelter practices and conditions. Secondly, we could not include board-certified veterinary behaviourists or welfare specialists in the current study, as such a system is yet to exist in Taiwan. We aimed to mitigate this limitation by inviting experts from diverse professional backgrounds and establishing specific criteria for expert qualifications. Thirdly, the reliance on the researchers’ network for expert recruitment may have introduced selection bias, potentially excluding other qualified professionals with differing viewpoints. Furthermore, this study employed a score-based Delphi method rather than the more common consensus-based Delphi approach. While the score-based method allowed for efficient evaluation of over 50 WCs, reaching a full consensus among experts may have provided more robust conclusions but would have required significantly more time. Additionally, although the experts ranked depressive disorders and fear-related and anxiety disorders as the most severe WCs, shelters in Taiwan lack sufficient resources to make such diagnoses. Thus, the results may deviate from reality, especially because there was no veterinary behavioural specialist in the expert group. Finally, some overlap among welfare concerns (e.g., “limited access to toys” and “insufficient sensory enrichment”) may have led to redundancy in the prioritisation process. Although expert discussions addressed these overlaps to some extent, further refinement of the WC list is needed in future studies.

## 5. Conclusions

This study identified 59 WCs affecting shelter dogs, with 49 considered critical for PASs in Taiwan based on expert opinions. The findings emphasise the importance of addressing environmental and behavioural interaction factors while considering both the severity and duration of WCs when assessing their welfare impact. The comprehensive WC list serves as a practical tool for evaluating shelter practices and guiding the allocation of resources to improve dog welfare. Future research should extend this framework to include dog populations in different geographical and shelter contexts and employ enhanced methods for quantifying welfare impacts. These efforts will advance our understanding of shelter dog welfare and support more effective strategies for improving the welfare of shelter dogs and dogs in general.

## Figures and Tables

**Table 1:** The final version of the welfare condition list for the dogs at public animal shelters in Taiwan.

Domain	Welfare Condition	Reference
Nutrition	Food spoilage	On-site observation
	2. Insufficient food supply (insufficient caloric intake)	[48]
	3. Insufficient water supply	[48]
	4. Water or food contamination by excrement of dogs	On-site observation
	5. Water or food contamination by excrement of rats	On-site observation
Physical Environment	Exposure to lower critical temperature (i.e., <10 °C or 50 °F)	[48,61]
	2. Exposure to upper critical temperature (i.e., >35 °C or 95 °F)	[48,61]
	3. Floor with gaps (such as non-slatted metal bar and wire-meshed floor)	[48]
	4. Inadequate wind-proofing	[48]
	5. Intermittent, excessive barking noise	[14]
	6. Lack of suitable substrate for resting (including soft, cloth, or elastic materials, etc.)	[48,62]
	7. Limited or no access to natural light	[61]
	8. Limited space (including being confined by crate space or overcrowding)	[27]
	9. Overstimulating conspecific and shelter odour	On-site observation
	10. Poor ventilation	[63]
	11. Space being restricted by a leash in the long term	On-site observation
	12. Wet floor (including cleansed floor or uncleansed floor with excrement)	[48,62]
Health	*Anaplasma phagocytophilum* infection (Canine granulocytic anaplasmosis) *	[64,65]
	2. *Anaplasma platys* infection (Canine thrombocytic anaplasmosis) *	[64,65]
	3. Babesiosis *	[66,67,68]
	4. Canine distemper *	[29,69]
	5. Canine infectious hepatitis *	[70]
	6. Canine infectious respiratory disease complexes *	[29,71]
	7. Canine leishmaniasis *	[68,72]
	8. Canine parvovirus infection *	[29,69]
	9. Cutaneous fungal infection	[73]
	10. Cutaneous mite infestation and/or infection (such as demodex, scabies, and otodectic mite)	[73,74]
	11. Depressive disorders (if there is no diagnosis, the judgement will be based on the occurrence of relevant symptoms)	[75,76]
	12. Dermatitis caused by excessive licking	[73,77]
	13. Dirofilariasis	[64,65,78]
	14. *Ehrlichia canis* infection *	[64,65,66,78]
	15. Enteric protozoal diseases (such as giardiasis and cryptosporidiosis) *	[79]
	16. Fear-related or anxiety disorders (if there is no diagnosis, the judgement can be based on the occurrence of relevant symptoms)	[31,76]
	17. Flea infestation	[73,80]
	18. Gastrointestinal nematode or tapeworm infestation and/or infection *	[79]
	19. Hepatozoonosis *	[64]
	20. Leptospirosis	[78]
	21. Lyme disease (borreliosis) *	[64,65,78]
	22. Operation without sufficient analgesia *	[48]
	23. Periodontal disease	[81,82]
	24. Rocky Mountain spotted fever *	[65,83]
	25. Tick infestation	[64,73]
	26. Tick paralysis *	[64,84]
	27. Traumatic injuries (caused by humans, dogs, or objects) with systemic symptoms	[48]
	28. Traumatic injuries (caused by humans, dogs, or objects) without systemic symptoms	[48]
Behavioural Interactions	Exposure to novel surroundings	[25,85]
	2. Fear, anxiety, or discomfort-inducing stimuli (including handling, interactions, sounds, etc.) made by staff and volunteers ^+^	[42,86]
	3. Fear, anxiety, or discomfort-inducing stimuli (including handling, interactions, sounds, etc.) made by visitors ^‡^	[42,86]
	4. Insufficient food diversity	[87]
	5. Insufficient foraging opportunities	[87]
	6. Insufficient opportunities for interactions (mainly social play) with non-cohabited dogs	On-site observation
	7. Insufficient outdoor activities	[88]
	8. Insufficient sensory enrichment (except food diversity)	[89,90,91]
	9. Lack of neutral to positive human companionship	[25,48,87]
	10. Lack of neutral to positive interactions (such as accompanying and social play) from cohabited dogs	[48,87]
	11. Limited access to toys	[90,92]
	12. Quarantine or isolation due to infectious diseases	[93]
	13. Single housing	[48,87]
	14. Unsuitable cohabitation with other dogs	[94,95]

* These conditions were answered by veterinarians only. ^+^ This condition was revised after the expert focus group discussion. ^‡^ This condition was added after the expert focus group discussion.

**Table 2:** The welfare conditions for dogs at public animal shelters in Taiwan with high prevalence, according to 14 Taiwanese experts.

Rank Order	Domain	Welfare Condition	Median (Interquartile Range)
1	Behavioural Interaction	Limited access to toys	4.00 (4.00–5.00)
	Physical Environment	Insufficient foraging opportunities	4.00 (4.00–5.00)
3	Behavioural Interaction	Intermittent, excessive barking noise	4.00 (3.25–4.75)
4	Behavioural Interaction	Lack of neutral to positive human companionship	4.00 (4.00–4.00)
5	Behavioural Interaction	Insufficient sensory enrichment (except food diversity)	4.00 (3.00–4.00)
	Physical Environment	Lack of suitable substrate for resting (including soft, cloth, or elastic materials, etc.)	4.00 (3.00–4.00)
	Behavioural Interaction	Insufficient food diversity	4.00 (3.00–4.00)
	Physical Environment	Wet floor (including cleansed floor or uncleansed floor with excrement)	4.00 (3.00–4.00)
9	Behavioural Interaction	Insufficient outdoor activities	3.50 (3.00–4.00)
10	Behavioural Interaction	Exposure to novel surroundings	3.00 (3.00–4.00)
	Behavioural Interaction	Insufficient opportunities for interactions (mainly social play) with non-cohabited dogs	3.00 (3.00–4.00)
12	Physical Environment	Overstimulating conspecific and shelter odour	3.00 (2.00–4.00)
	Behavioural Interaction	Fear, anxiety, or discomfort-inducing stimuli (including handling, interactions, sounds, etc.) made by staff and volunteers	3.00 (2.00–4.00)
	Physical Environment	Limited space (including being confined by crate space or overcrowding)	3.00 (2.00–4.00)
15	Behavioural Interaction	Fear, anxiety, or discomfort-inducing stimuli (including handling, interactions, sounds, etc.) made by visitors	3.00 (2.00–3.75)
16	Physical Environment	Limited or no access to natural light	3.00 (1.00–3.00)

**Table 3:** The conditions with high negative welfare impact on dogs at the individual level at public animal shelters in Taiwan, according to 14 Taiwanese experts.

Rank Order	Domain	Welfare Condition	Median (Interquartile Range)
1	Health	Depressive disorders (if there is no diagnosis, the judgement can be based on the occurrence of relevant symptoms)	16.00 (13.50–18.00)
2	Health	Fear-related or anxiety disorders (if there is no diagnosis, the judgement can be based on the occurrence of relevant symptoms)	15.00 (9.00–20.00)
3	Physical Environment	Limited space (including being confined by crate space or overcrowding)	15.00 (8.25–19.00)
4	Physical Environment	Intermittent, excessive barking noise	12.50 (9.00–15.00)
5	Health	Canine leishmaniasis	12.00 (8.00–12.00)
6	Health	Periodontal disease	11.00 (9.00–15.00)
7	Behavioural Interaction	Insufficient outdoor activities	11.00 (8.00–15.00)
8	Physical Environment	Floor with gaps (such as non-slatted metal bar and wire-meshed floor)	11.00 (5.00–14.25)
9	Physical Environment	Poor ventilation	11.00 (4.50–14.25)
10	Behavioural Interaction	Insufficient sensory enrichment (except food diversity)	10.00 (9.00–15.00)
11	Physical Environment	Overstimulating conspecific and shelter odour	10.00 (4.50–14.25)
12	Behavioural Interaction	Unsuitable cohabitation with other dogs	9.50 (6.00–12.00)
13	Behavioural Interaction	Limited access to toys	9.50 (5.00–10.00)
14	Physical Environment	Limited or no access to natural light	9.00 (4.00–16.00)
15	Behavioural Interaction	Fear, anxiety, or discomfort-inducing stimuli (including handling, interactions, sounds, etc.) made by staff and volunteers	9:00 (5.25–14.25)
16	Health	*Anaplasma platys* infection (Canine thrombocytic anaplasmosis)	9.00 (8.00–12.00)
17	Health	Cutaneous mite infestation and/or infection (such as demodex, scabies, and otodectic mite)	9.00 (6.00–12.00)
	Health	Babesiosis	9.00 (6.00–12.00)
19	Health	*Ehrlichia canis* infection	9.00 (6.00–9.00)
20	Health	Dermatitis caused by excessive licking	9.00 (4.00–10.00)

## Data Availability

The original contributions presented in this study are included in this article. Further enquiries can be directed to the corresponding author.

## Appendix A

**Table A1:** The 49 important welfare conditions list for the dogs at public animal shelters in Taiwan.

Domain	Welfare Condition
Nutrition	Food spoilage
	2. Insufficient food supply (insufficient caloric intake)
	3. Insufficient water supply
	4. Water or food contamination by excrement of rats
Physical Environment	Exposure to lower critical temperature (i.e., <10 °C or 50 °F)
	2. Exposure to upper critical temperature (i.e., >35 °C or 95 °F)
	3. Floor with gaps (such as non-slatted metal bar and wire-meshed floor)
	4. Intermittent, excessive barking noise
	5. Lack of suitable substrate for resting (including soft, cloth, or elastic materials, etc.)
	6. Limited or no access to natural light
	7. Limited space (including being confined by crate space or overcrowding)
	8. Overstimulating conspecific and shelter odour
	9. Poor ventilation
	10. Space being restricted by a leash in the long term
	11. Wet floor (including cleansed floor or uncleansed floor with excrement)
Health	*Anaplasma phagocytophilum* infection (Canine granulocytic anaplasmosis)
	2. *Anaplasma platys* infection (Canine thrombocytic anaplasmosis)
	3. Babesiosis
	4. Canine distemper
	5. Canine infectious hepatitis
	6. Canine leishmaniasis
	7. Canine parvovirus infection
	8. Cutaneous fungal infection
	9. Cutaneous mite infestation and/or infection (such as demodex, scabies, and otodectic mite)
	10. Depressive disorders (if there is no diagnosis, the judgement will be based on the occurrence of relevant symptoms)
	11. Dermatitis caused by excessive licking
	12. Dirofilariasis
	13. Enteric protozoal diseases (such as giardiasis and cryptosporidiosis)
	14. *Ehrlichia canis* infection
	15. Fear-related or anxiety disorders (if there is no diagnosis, the judgement can be based on the occurrence of relevant symptoms)
	16. Hepatozoonosis
	17. Leptospirosis
	18. Lyme disease (borreliosis)
	19. Operation without sufficient analgesia
	20. Periodontal disease
	21. Rocky Mountain spotted fever
	22. Tick paralysis
	23. Traumatic injuries (caused by humans, dogs, or objects) with systemic symptoms
Behavioural Interactions	Exposure to novel surroundings
	2. Fear, anxiety, or discomfort-inducing stimuli (including handling, interactions, sounds, etc.) made by staff and volunteers
	3. Fear, anxiety, or discomfort-inducing stimuli (including handling, interactions, sounds, etc.) made by visitors
	4. Insufficient food diversity
	5. Insufficient foraging opportunities
	6. Insufficient opportunities for interactions (mainly social play) with non-cohabited dogs
	7. Insufficient outdoor activities
	8. Insufficient sensory enrichment (except food diversity)
	9. Lack of neutral to positive human companionship
	10. Limited access to toys
	11. Unsuitable cohabitation with other dogs

**Table A2:** This study recommends the methods for evaluating each welfare condition.

Domain	Welfare Condition	Assessment Method
Nutrition	Food spoilage	Observation
	2. Insufficient food supply (insufficient caloric intake)	Observation, staff interviews, and review of medical records.
	3. Insufficient water supply	Observation
	4. Water or food contamination by excrement of dogs	Observation
	5. Water or food contamination by excrement of rats	Observation, staff interviews
Physical Environment	Exposure to lower critical temperature (i.e., <10 °C or 50 °F)	Instrumental measurement, government open data
	2. Exposure to upper critical temperature (i.e., >35 °C or 95 °F)	Instrumental measurement, government open data
	3. Floor with gaps (such as non-slatted metal bar and wire-meshed floor)	Observation
	4. Inadequate wind-proofing	Observation
	5. Intermittent, excessive barking noise	Instrumental measurement
	6. Lack of suitable substrate for resting (including soft, cloth, or elastic materials, etc.)	Observation
	7. Limited or no access to natural light	Instrumental measurement
	8. Limited space (including being confined by crate space or overcrowding)	Observation
	9. Overstimulating conspecific and shelter odour	Instrumental measurement
	10. Poor ventilation	Instrumental measurement
	11. Space being restricted by a leash in the long term	Observation
	12. Wet floor (including cleansed floor or uncleansed floor with excrement)	Observation, staff interviews
Health	*Anaplasma phagocytophilum* infection (Canine granulocytic anaplasmosis)	Review of medical records, staff interviews
	2. *Anaplasma platys* infection (Canine thrombocytic anaplasmosis)	Review of medical records, staff interviews
	3. Babesiosis	Review of medical records, staff interviews
	4. Canine distemper	Review of medical records, staff interviews
	5. Canine infectious hepatitis	Review of medical records, staff interviews
	6. Canine infectious respiratory disease complexes	Review of medical records, staff interviews
	7. Canine leishmaniasis	Review of medical records, staff interviews
	8. Canine parvovirus infection	Review of medical records, staff interviews
	9. Cutaneous fungal infection	Review of medical records, staff interviews
	10. Cutaneous mite infestation and/or infection (such as demodex, scabies, and otodectic mite)	Review of medical records, staff interviews
	11. Depressive disorders (if there is no diagnosis, the judgement will be based on the occurrence of relevant symptoms)	Review of medical records, staff interviews
	12. Dermatitis caused by excessive licking	Review of medical records, staff interviews
	13. Dirofilariasis	Review of medical records, staff interviews
	14. *Ehrlichia canis* infection	Review of medical records, staff interviews
	15. Enteric protozoal diseases (such as giardiasis and cryptosporidiosis)	Review of medical records, staff interviews
	16. Fear-related or anxiety disorders (if there is no diagnosis, the judgement can be based on the occurrence of relevant symptoms)	Review of medical records, staff interviews
	17. Flea infestation	Review of medical records, staff interviews
	18. Gastrointestinal nematode or tapeworm infestation and/or infection	Review of medical records, staff interviews
	19. Hepatozoonosis	Review of medical records, staff interviews
	20. Leptospirosis	Review of medical records, staff interviews
	21. Lyme disease (borreliosis)	Review of medical records, staff interviews
	22. Operation without sufficient analgesia	Review of medical records, staff interviews
	23. Periodontal disease	Review of medical records, staff interviews
	24. Rocky Mountain spotted fever	Review of medical records, staff interviews
	25. Tick infestation	Review of medical records, staff interviews
	26. Tick paralysis	Review of medical records, staff interviews
	27. Traumatic injuries (caused by humans, dogs, or objects) with systemic symptoms	Observation, review of medical records, staff interviews
	28. Traumatic injuries (caused by humans, dogs, or objects) without systemic symptoms	Observation, review of medical records, staff interviews
Behavioural Interactions	Exposure to novel surroundings	Review of medical records, staff interviews
	2. Fear, anxiety, or discomfort-inducing stimuli (including handling, interactions, sounds, etc.) made by staff and volunteers	Observation, staff interviews
	3. Fear, anxiety, or discomfort-inducing stimuli (including handling, interactions, sounds, etc.) made by visitors	Observation
	4. Insufficient food diversity	Observation, staff interviews
	5. Insufficient foraging opportunities	Observation, staff interviews
	6. Insufficient opportunities for interactions (mainly social play) with non-cohabited dogs	Observation, staff interviews
	7. Insufficient outdoor activities	Observation, staff interviews
	8. Insufficient sensory enrichment (except food diversity)	Observation, staff interviews
	9. Lack of neutral to positive human companionship	Observation, staff interviews
	10. Lack of neutral to positive interactions (such as accompanying and social play) from cohabited dogs	Observation, staff interviews
	11. Limited access to toys	Observation
	12. Quarantine or isolation due to infectious diseases	Observation
	13. Single housing	Observation, staff interviews
	14. Unsuitable cohabitation with other dogs	Observation, staff interviews
